# Engineering a fungal peroxidase that degrades lignin at very acidic pH

**DOI:** 10.1186/1754-6834-7-114

**Published:** 2014-07-24

**Authors:** Elena Fernández-Fueyo, Francisco J Ruiz-Dueñas, Angel T Martínez

**Affiliations:** Centro de Investigaciones Biológicas, CSIC, Ramiro de Maeztu 9, E-28040 Madrid, Spain; Department of Biotechnology, TU Delft, Julianalaan 136, 2628 BL Delft, Netherlands

**Keywords:** Acidic pH stability, Catalytic tryptophan, Lignin model dimer, Manganese peroxidase, Versatile peroxidase, White-rot fungal genomes

## Abstract

**Background:**

Ligninolytic peroxidases are divided into three families: manganese peroxidases (MnPs), lignin peroxidases (LiPs), and versatile peroxidases (VPs). The latter two are able to degrade intact lignins, as shown using nonphenolic lignin model compounds, with VP oxidizing the widest range of recalcitrant substrates. One of the main limiting issues for the use of these two enzymes in lignocellulose biorefineries (for delignification and production of cellulose-based products or modification of industrial lignins to added-value products) is their progressive inactivation under acidic pH conditions, where they exhibit the highest oxidative activities.

**Results:**

In the screening of peroxidases from basidiomycete genomes, one MnP from *Ceriporiopsis subvermispora* was found to have a remarkable acidic stability. The crystal structure of this enzyme recently became available and, after comparison with *Pleurotus ostreatus* VP and *Phanerochaete chrysosporium* LiP structures, it was used as a robust scaffold to engineer a stable VP by introducing an exposed catalytic tryptophan, with different protein environments. The variants obtained largely maintain the acidic stability and strong Mn^2+^-oxidizing activity of the parent enzyme, and the ability to oxidize veratryl alcohol and Reactive Black 5 (two simple VP substrates) was introduced. The engineered peroxidases present more acidic optimal pH than the best VP from *P. ostreatus*, enabling higher catalytic efficiency oxidizing lignins, by lowering the reaction pH, as shown using a nonphenolic model dimer.

**Conclusions:**

A peroxidase that degrades lignin at very acidic pH could be obtained by engineering an exposed catalytic site, able to oxidize the bulky and recalcitrant lignin polymers, in a different peroxidase type selected because of its high stability at acidic pH. The potential of this type of engineered peroxidases as industrial biocatalysts in lignocellulose biorefineries is strongly enhanced by the possibility to perform the delignification (or lignin modification) reactions under extremely acidic pH conditions (below pH 2), resulting in enhanced oxidative power of the enzymes.

## Background

Lignin removal represents the limiting step for the use of renewable plant biomass in lignocellulose biorefineries for the development of a sustainable bioeconomy [1]. Only a group of basidiomycetes, called white-rot fungi, are generally considered as efficient lignin degraders in nature [2], although the ability to degrade lignin has also been claimed for a range of soil bacteria [3, 4]. According to genomic data, ligninolytic peroxidases - lignin peroxidase (LiP, EC1.11.1.14), manganese peroxidase (MnP, EC1.11.1.13), and versatile peroxidase (VP, EC 1.11.1.16) - are exclusive to white-rot basidiomycetes, and would play a central role in lignin biodegradation [5]. Due to the high redox potential required for lignin degradation, these enzymes are of high industrial interest for (i) delignification and production of bioethanol and other cellulose-based chemicals in lignocellulose biorefineries, (ii) modification of lignins from the bioethanol and paper pulp sectors for the production of added-value products such as dispersants, adhesives, and aromatic chemicals, and (iii) use in a variety of green chemistry and bioremediation reactions of interest [2, 6].

The LiP and MnP families were described in the model white-rot basidiomycete *Phanerochaete chrysosporium* [7, 8]. VP, the third family of ligninolytic peroxidases, was described later in *Pleurotus eryngii* [9, 10] and is also found in *Bjerkandera* species [11] and other basidiomycetes. LiPs possess a tryptophan residue at the enzyme surface, corresponding to Trp^171^ in *P. chrysosporium* LiP (isoenzyme H8), that enables direct oxidation of lignin via long-range electron transfer to heme [12, 13]. MnPs possess three residues located in an anionic pocket (Glu^35^, Glu^39^, and Asp^179^ in *P. chrysosporium* isoenzyme MnP1) forming a Mn-binding site [14], in which Mn^2+^ is oxidized to Mn^3+^. VPs possess both a catalytic tryptophan (Trp^164^ in *P. eryngii* isoenzyme VPL) and a manganese-binding site (VPL Glu^36^, Glu^40^, and Asp^175^) [15]. Moreover, a low-efficiency site involved in the oxidation of phenols operates at the entrance of the heme distal pocket of VP [16]. On the other hand, the oxidation of phenols and dyes in the absence of manganese has been described in *Pleurotus ostreatus* MnPs [17] belonging to the subfamily of short MnPs defined in genomic screenings together with the so-called long and extralong MnP subfamilies [5]. In addition to its ability to oxidize Mn^2+^ to Mn^3+^ (as MnP) and lignin (as LiP), VP oxidizes high redox potential aromatics and dyes, such as Reactive Black 5 (RB5), which LiP oxidizes only in the presence of redox mediators [15]. This wider VP activity would be related to differences in the residues surrounding the exposed catalytic tryptophan which are not conserved between LiP and VP.

Wild peroxidases are not well suited for industrial use, which often requires particular substrate specificities and application conditions (including pH, temperature, and reaction media) in addition to high production levels. Therefore, protein engineering is often required to obtain highly produced and efficient biocatalysts [6]. In nature, wood lignin degradation takes place at acidic pH due to the secretion of organic acids by white-rot basidiomycetes [18] in agreement with the highest LiP and VP activities on aromatic compounds, which optimally takes place at pH ≤ 3 [2]. Although partial stability under acidic conditions is, therefore, a requisite for enzymatic ligninolysis, inactivation inexorably takes place at acidic pH. Namely, LiP (from *P. chrysosporium*) and VP (from *P. eryngii* and a *Bjerkandera* sp.) are known to lose all the activity at pH below 4.0 in less than 5 h (at 25°C) [19, 20]. The lack of higher stability under acidic pH thus limits the industrial use of these enzymes, because the reactions must be carried out under pH conditions in which the enzymes are less active, to protect them from inactivation. Similarly, improving the oxidative stability of these and other peroxidases against H_2_O_2_ is also a prerequisite for industrial applicability [21].

In this study, we screened ligninolytic peroxidases for stability at acidic pH based on 14 genes from the genomes of the white-rot fungi *P. ostreatus* and *Ceriporiopsis subvermispora* available at the Joint Genome Institute (of the US Department of Energy). Gene duplication in basidiomycete genomes results in a variety of isoenzymes (up to 6 and 13 MnPs in *P. ostreatus* and *C. subvermispora*, respectively) whose differential expression/production under variable environmental conditions has been investigated by quantitative PCR and liquid chromatography coupled to MS/MS [22]. Among them, *C. subvermispora* MnP6 showed a remarkable acidic stability, being the most acidic-pH stable ligninolytic peroxidase described to date. This peroxidase isoenzyme was used as a robust protein scaffold to obtain a VP-type peroxidase by introducing an exposed catalytic tryptophan, in two different protein environments. The engineered peroxidase would be of interest as an industrial biocatalyst, because of the combination of its promiscuity in oxidizing different recalcitrant aromatic compounds and dyes, and its high stability at acidic pH, improving its action on lignins.

## Results

### Screening for peroxidases highly stable at acidic pH

Fourteen peroxidases from the genomes of *P. ostreatus* (nine VP or MnP encoding genes) and *C. subvermispora* (five MnP encoding genes) were obtained by *Escherichia coli* overexpression of the mature protein codifying sequences, followed by *in vitro* activation (for cofactor and Ca^2+^ incorporation, and disulfide bond formation) and purification to electrophoretic homogeneity in a single chromatographic step. In this way, 35 to 55 mg of total recombinant peroxidase protein were obtained from the inclusion bodies recovered from 1 L of culture, which resulted in 1.5 to 14 mg of pure active peroxidase after *in vitro* activation (active protein representing 3 to 28% of the total protein) and purification (pure protein representing 85 to 95% of the active protein). The molecular masses from sodium dodecyl sulfate-polyacrylamide gel electrophoresis (SDS-PAGE) coincided with the predicted protein sequences, and the electronic absorption spectra showed the Soret (406 nm) and other typical bands of the peroxidase resting state, confirming that the heme cofactor was correctly incorporated.

**Figure 1:**
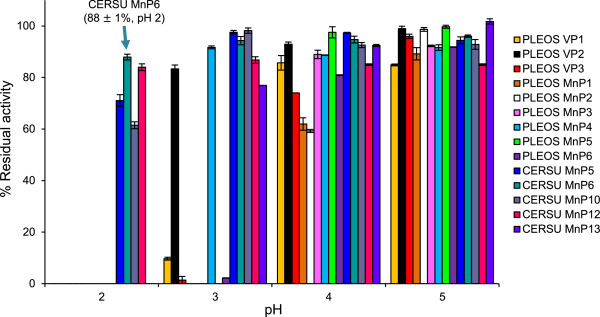
**Acidic pH stabilities of 14 ligninolytic peroxidases from the** ***P. ostreatus*** **and** ***C. subvermispora*** **genomes.** Residual activities of *P. ostreatus* (PLEOS) VP1 (PC9 genome model 137757), VP2 (PC15 genome model 1113241), VP3 (PC15 genome model 156336), short MnP1 (PC15 genome model 1096331), short MnP2 (PC15 genome model 199510), short MnP3 (PC15 genome model 1089546), short MnP4 (PC15 genome model 1099081), short MnP5 (PC15 genome model 199511), and short MnP6 (PC15 genome model 1041740) and *C. subvermispora* (CERSU) extralong MnP5 (genome model 49683), extralong MnP6 (genome model 50686), long MnP10 (genome model 117436), long MnP12 (genome model 157986), and short MnP13 (genome model 124076), after 24-h incubation at 4°C, in 100 mM Britton-Robinson (BR) buffer of pH 2.0 to 5.0. Means and 95% confidence limits from triplicate experiments.

The pH stability of the different enzymes was estimated as the residual activity after 24-h incubation at 4°C, and the results obtained in the range of pH 2.0 to 5.0 are shown in Figure 1. The 14 peroxidases are fully stable at pH 5.0 (80 to 100% of the maximal residual activity, generally obtained at pH 6.0); however, they present huge differences in their stability towards acidic pH. Several of them started to be inactivated at pH 4.0, half of them were nearly completely inactivated at pH 3.0, and only four maintained their activity after incubation at pH 2.0. These results showed the remarkable acidic stability of the long and extralong MnPs from *C. subvermispora*, which maintained over 60% and 70% of the initial activity at pH 2.0, respectively, the extralong MnP6 being the most stable isoenzyme (arrow in Figure 1). On the other hand, none of the *P. ostreatu*s peroxidases (VPs and short MnPs) were stable at pH 2.0.

### Engineering a catalytic tryptophan on an acidic-pH stable extralong MnP

**Figure 2:**
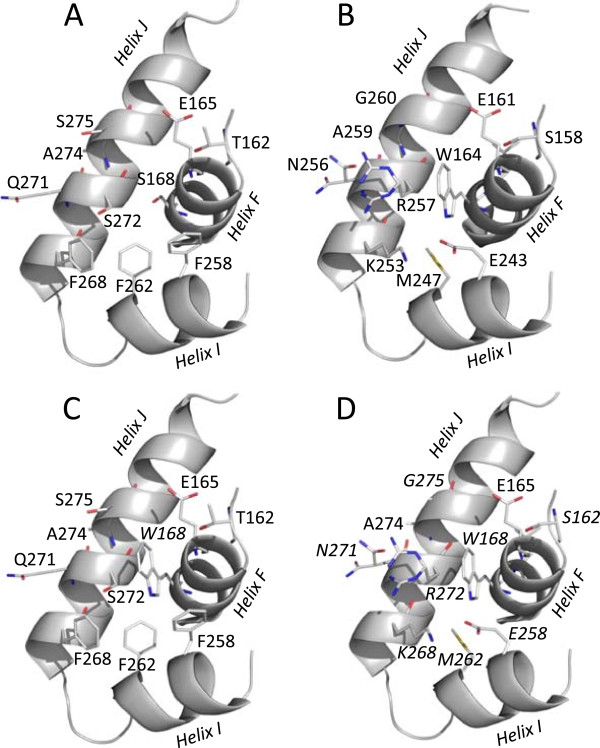
**Engineering the acidic-pH stable MnP6 from** ***C. subvermispora*** **by introducing a catalytic tryptophan (S168W variant) and changing the tryptophan environment (S168W-environment variant).** The two variants were built by substituting Ser^168^ of *C. subvermispora* MnP6 **(A)** by a catalytic tryptophan to obtain the S168W variant **(C)** and engineering its environment as found in *P. ostreatus* VP1 **(B)** to obtain the S168W-environment variant **(D)**. The mutated residues in **C** (Trp^168^) and **D** (Ser^162^, Trp^168^, Glu^258^, Met^262^, Lys^268^, Asn^271^, Arg^272^, and Gly^275^) are in italics. Based on MnP6 (4CZN) and VP1 (4BLK) crystal structures, and homology models of the S168W and S168W-environment variants.

The crystal structures of the *E. coli*-produced *P. ostreatus* VP1 (4BLK) and *C. subvermispora* MnP6 (4CZN) were recently solved. The *C. subvermispora* MnP6 presents a serine, Ser^168^ (Figure 2A), at the position of the VP1 catalytic Trp^164^ (Figure 2B), and two approaches were followed to confer VP-type activity on aromatic substrates and dyes to this enzyme by protein engineering. The first strategy consisted in entering only the tryptophan residue (Figure 2C) to obtain the S168W variant, while the second approach included modeling a tryptophan environment similar to that of Trp^164^ in *P. ostreatus* VP1, resulting in the S168W-environment variant (Figure 2D).

**Figure 3:**
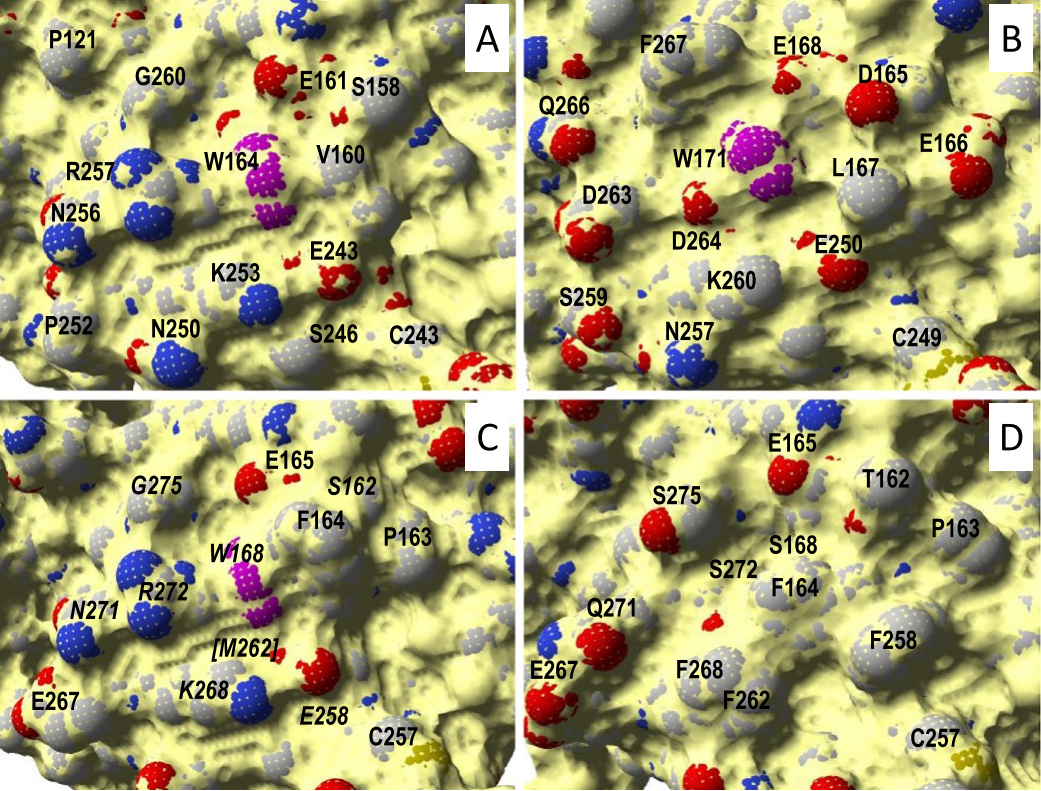
**Details of surface environment of the catalytic tryptophan in** ***P. ostreatus*** **VP1 (A),** ***P. chrysosporium*** **LiP-H8 (B), and S168W-environment variant of** ***C. subvermispora*** **MnP6 (C), compared with the same region in the native** ***C. subvermispora*** **MnP6 (D).** Solvent access surface shown as yellow, tryptophan residues (VP1, LiP-H8, and S168W-environment Trp^164^, Trp^171^, and Trp^168^, respectively) as magenta, and other residues as CPK-colored van der Waals spheres. The mutated residues in **C** are in italics. From 4BLK **(A)**, 1LGA **(B)**, 4CZN **(D)**, and homology model of the SW168-environment variant based on 4BLK **(C)**.

To design the second MnP6 variant, we compared the catalytic tryptophan surface environments in the *P. ostreatus* VP1 and *P. chrysosporium* LiP-H8 (1LGA) crystal structures (Figure 3A and B), and the corresponding region in the *C. subvermispora* MnP6 structure (Figure 3D). In this way, we selected those residues that could contribute to the higher promiscuity of VP in oxidizing aromatic substrates and dyes (compared with LiP) for their introduction in *C. subvermispora* MnP6. The environment of VP1 Trp^164^ is less electronegative including only the acidic Glu^161^ and Glu^243^, whereas the LiP-H8 Trp^171^ is surrounded by Asp^165^, Glu^166^, Glu^168^, Glu^250^, and Asp^264^. Moreover, the basic Lys^253^ and Arg^257^ exist in the VP tryptophan environment, whereas only Lys^260^ is present in the LiP tryptophan environment. On the other hand, the VP tryptophan is surrounded by small residues, such as Gly^260^ and Val^160^, instead of the bulky Leu^167^ and Phe^267^ in LiP. Finally, Asn^257^ and Met^247^ are conserved among VPs, forming hydrogen bonds with Arg^247^ and Glu^243^, respectively, but they are absent from LiP. According to the above analysis, the following mutations were performed in MnP6 to introduce the exposed catalytic tryptophan and provide it with a VP-type environment (S168W-environment variant): T162S, S168W, F258E, F262M, F268K, Q271N, S272R, and S275G (Figures 2D and 3C).

The two variants were successfully overproduced in *E. coli*, *in vitro* activated, and purified.

### Catalytic properties of the extralong MnP variants with a putative catalytic tryptophan

After obtaining the S168W and S168W-environment variants of the acidic-pH stable *C. subvermispora* MnP6, we investigated their catalytic properties to learn the effect of the mutations included. Table 1 shows their kinetic constants, compared with those of native *C. subvermispora* MnP6, *P. ostreatus* VP1, and *P. chrysosporium* LiP-H8, also produced in *E. coli*. For this comparison we used (i) Mn^2+^, which is oxidized at the MnP and VP Mn oxidation site, (ii) high redox potential veratryl alcohol (VA) and RB5, which are oxidized at the LiP and VP catalytic tryptophan, and (iii) low redox potential 2,2'-azino-bis(3-ethylbenzothiazoline-6-sulfonate) (ABTS) and 2,6-dimethoxyphenol (DMP), which are oxidized by VPs at the catalytic tryptophan (with high efficiency) but also at a second (low-efficiency) site [15, 16]. The two variants conserve the high catalytic efficiency of extralong MnP6 oxidizing Mn^2+^, with a *K*_m_ value approximately tenfold lower than that of VP1, demonstrating that the mutations introduced did not affect the Mn^2+^-binding ability. More importantly, the kinetic constants showed that the two strategies for introducing a functional catalytic tryptophan were basically successful, since both variants incorporated the ability to oxidize VA as well as RB5.

**Table 1:** **Kinetic constants for oxidation of five substrates (at fixed pH for each substrate) by** ***C. subvermispora*** **MnP6 and its S168W and S168W-environment variants compared with** ***P. chrysosporium*** **LiP-H8** and ***P. ostreatus*** **VP1**

		MnP6	S168W	S168W-environment	LiP-H8	VP1
Mn^2+^	*K*_*m*_ (μM)	8.7 ± 1.6	8.5 ± 1.2	11.0 ± 1.8	-	98.0 ± 5.6
	*k*_cat_ (s^-1^)	83.0 ± 5.0	60.5 ± 5.0	66.9 ± 6.0	0	185.0 ± 2.6
	*k*_cat_/*K*_*m*_ (s^-1^.mM^-1^)	9540 ± 650	7110 ± 650	6060 ± 650	0	1900 ± 90
VA	*K*_*m*_ (μM)	0	2740 ± 560	21600 ± 200	190 ± 17	5500 ± 46
	*k*_cat_ (s^-1^)	0	0.54 ± 0.04	1.69 ± 0.04	17.5 ± 0.5	12.7 ± 0.5
	*k*_cat_/*K*_*m*_ (s^-1^ **.**mM^-1^)	-	0.197 ± 0	0.078 ± 0	92.0 ± 6.0	2.3 ± 0.2
RB5	*K*_*m*_ (μM)	0	12.6 ± 3	5.4 ± 3	0	5.4 ± 0.2
	*k*_cat_ (s^-1^)	0	7.8 ± 1.1	7.0 ± 1.1	0	12.9 ± 0.3
	*k*_cat_/*K*_*m*_ (s^-1^ **.**mM^-1^)	-	619 ± 27	1310 ± 100	-	2380 ± 50
ABTS	*K*_*m*_ (μM)	0	60.2 ± 13.4	0	23.0 ± 1.6	4.0 ± 0.4
	*k*_cat_ (s^-1^)	0	1.4 ± 0.1	0	13.0 ± 0.2	14.4 ± 0.4
	*k*_cat_/*K*_*m*_ (s^-1^ **.**mM^-1^)	-	23 ± 3	-	563 ± 36	3600 ± 20
DMP	*K*_*m*_ (μM)	0	0	0	5.8 ± 0.5	54 ± 4
	*k*_cat_ (s^-1^)	0	0	0	10.0 ± 0.1	6.6 ± 0.1
	*k*_cat_/*K*_*m*_ (s^-1^ **.**mM^-1^)	-	-	-	1720 ± 153	122 ± 7

Means and 95% confidence limits from triplicate reactions of the *C. subvermispora* MnP6 (JGI genome model 50686), its S168W and S168W-environment variants (the latter combining T162S, S168W, F258E, F262M, F268K, Q271N, S272R, and S275G mutations), *P. ostreatus* VP1 (JGI genome model 137757), and *P. chrysosporium* LiP-H8 (JGI genome model 131707) at 25°C in 0.1 M tartrate, pH 5.0 for Mn^2+^, pH 3.0 for VA, and pH 3.5 for RB5, DMP, and ABTS oxidation. ABTS and DMP oxidation by VP1 shows biphasic kinetics enabling calculation of a second low-efficiency set of constants (not shown).

Although the catalytic efficiency of the variants oxidizing VA was low compared with that of *P. chrysosporium* LiP-H8, it was more similar to that of *P. ostreatus* VP1. These differences are mainly due to the very different *K*_m_ values, which for LiP-H8 were in the micromolar range (*K*_m_ 190 ± 17 μM) while they were in the millimolar range for the other peroxidases, attaining the highest value for the S168W-environment variant (*K*_m_ 21.6 ± 0.2 mM). Interestingly, the S168W variant had a lower *K*_m_ value for VA (2.74 ± 0.56 mM) than VP1 (5.50 ± 0.05 mM), and a higher catalytic efficiency on this substrate than the S168W-environment variant.

Concerning RB5 oxidation, the two variants behave as VPs, being able to oxidize this recalcitrant dye, which was not oxidized by LiP-H8. When compared with *P. ostreatus* VP1, the variants present much lower differences in the catalytic efficiency than found for VA oxidation, and the highest efficiency corresponded to the S168W-environment variant. Since both variants showed similar activities on RB5 (*k*_cat_ 7 to 8 ± 1 s^-1^), the latter difference resulted in a lower *K*_m_ value of the S168W-environment variant for the dye, which was similar to that found for VP1 (5.4 μM).

Neither the extralong MnP6 nor the two mutated variants were able to oxidize DMP, as both VP1 and LiP-H8 did, suggesting that the engineered sites were unable to properly bind this phenolic substrate. Similarly, the S168-environment variant did not oxidize ABTS, and the S168W variant showed lower catalytic efficiency than VP1 and LiP-H8.

### Acidic stability of the VP-type variants of the extralong MnP

**Figure 4:**
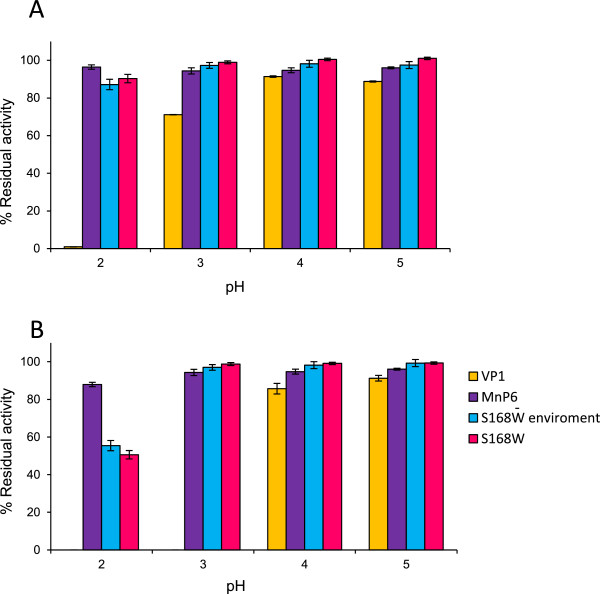
**pH stabilities of the S168W and S168W-environment variants of** ***C. subvermispora*** **MnP6 compared to the native MnP6 and the** ***P. ostreatus*** **VP1 after 4-h (A) and 24-h incubation (B).** Residual activities were determined by 1.5 mM Mn^2+^ oxidation in 0.1 M tartrate, pH 5.0, after incubation at 4°C, in 100 mM BR buffer, pH 2.0 to 5.0. Means and 95% confidence limits from triplicate experiments.

After confirming that the two MnP6 variants (S168W and S168-environment) presented VP-type catalytic properties, we investigated their pH stabilities to determine if they retained the acidic-pH stability of the parent enzyme. With this purpose, their residual activities were estimated after incubating them, together with *C. subvermispora* MnP6 and *P. ostreatus* VP1, in the range of pH 1.6 to 8.0, at 4°C, and normalized as described above. The residual activities in the acidic-pH range of interest, after 4-h and 24-h incubation, are shown in Figure 4A and B, respectively.

At pH 4.0 and 5.0, the S168W and S168W-environment variants and the native *C. subvermispora* MnP6 showed 90 to 100% residual activities after both 4 and 24 h of incubation, while VP1 showed about 80% activity. However, at pH 3.0 a strong difference between the *C. subvermispora* MnP6, its directed variants, and the *P. ostreatus* VP1 was observed. This was especially noticeable after 24-h incubation, where VP1 lost over 90% activity, while the three other peroxidases retained about 90% of their activity. Finally, at pH 2.0, VP1 was fully inactivated after only 4 h, while significant inactivation of the MnP enzymes was only produced after 24-h incubation (always with residual activities >50%). Under the latter conditions, the S168W and S168W-environment variants retained most of the acidic-pH stability of the native *C. subvermispora* MnP6 (50 to 55% compared with about 70% residual activities, respectively), resulting in two VP-type enzymes with much higher pH stability than that of the natural VPs (such as *P. ostreatus* VP1, which is fully inactivated at this pH).

### Effect of pH on the activity and kinetic constants of the VP-type variants

Due to the high stability of the *C. subvermispora* MnP6 variants at acidic pH compared to VP, we investigated if their optimal pH for oxidation of VA and RB5 was lower than that normally used for VA and RB5 (pH 3.0 to 3.5) oxidation by VP and LiP (as shown, for example, in Table 1). To estimate the optimal pH for oxidation of these two substrates by the S168W and S168W-environment variants, we used saturating concentrations of VA and RB5 in BR buffer of pH 1.6 to 5.0.

**Figure 5:**
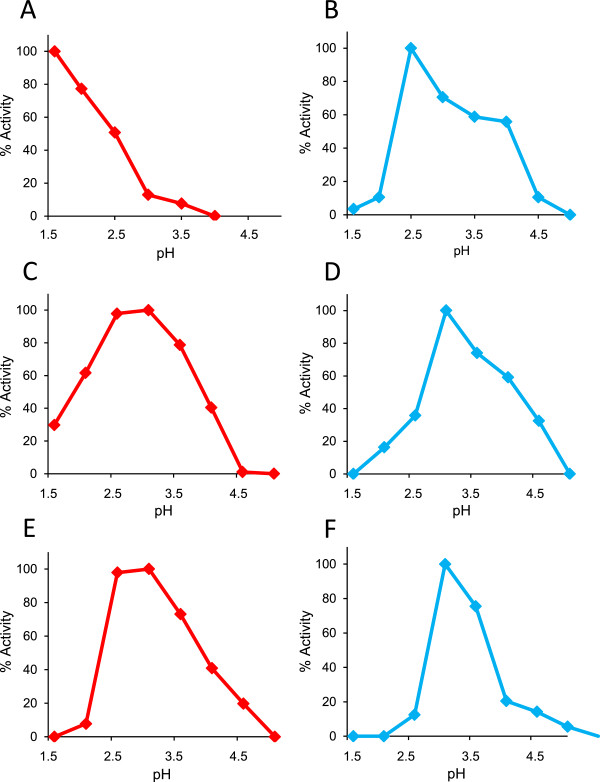
**Optimal pH for VA (left) and RB5 (right) oxidation by S168W (A and B) and S168W-environment (C and D) variants of** ***C. subvermispora*** **MnP6, and native** ***P. ostreatus*** **VP1 (E and F).** Activities for 6 mM VA **(A,** **C,** **and** **E)** and 15 μM RB5 **(B,** **D, and** **F)** oxidation by the S168W **(A and** **B)** and S168W-environment **(C and** **D)** variants, and native VP1 **(E and** **F)** were estimated in 100 mM BR buffer of pH 1.6 to 5.0. Means from triplicate experiments.

Optimal VA oxidation by the S168W variant took place under the most acidic conditions assayed, that is, at pH 1.6 (Figure 5A) (where the variant retained 50% residual activity after 24-h incubation at 4°C). With respect to pH 3.0 used in the standard assay, we found that the activity increased over fourfold, sixfold, and eightfold when the reaction was measured at pH 2.5, 2.0, and 1.6, respectively. On the other hand, optimal RB5 oxidation was at pH 2.5 (Figure 5B), the activity being 1.7-fold higher than at pH 3.5 (used in the standard assay) and over 28-fold higher than at the optimal pH for VA oxidation (pH 1.6). When these optimal pH values were compared with those of *P. ostreatus* VP1 (Figure 5E and F), a clear displacement to more acidic pH optima was observed for both VA and RB5 oxidation by the MnP6 variant. However, the S168W-environment variant did not show such displacement in the VA (Figure 5C) and RB5 (Figure 5D) oxidation optima. Since both variants are relatively stable at pH 1.6 (around 50% residual activity after 24 h), the less acidic optimal pH of the S168W-environment variant oxidizing VA (and RB5) is explained by the presence of a VP-type surface environment around the introduced catalytic tryptophan.

To obtain more information about the effect of pH on the oxidation of VA and RB5 by the S168W variant, whose optimal pH had been displaced to more acidic values, its kinetic constants were estimated at different pH values. A more than tenfold increase of the catalytic efficiency for VA oxidation was found when the reaction was performed at pH 1.6, instead of at pH 3.0 (Table 2, top). Interestingly, this increase resulted in improvements of both *K*_m_ (changing from 2.7 ± 0.6 mM to 0.7 ± 0.1 mM) and turnover (*k*_cat_ increasing from 0.5 ± 0.0 s^-1^ to 1.4 ± 0.1 s^-1^) values at the most acidic pH. Under these conditions, the catalytic efficiency of the variant (2.1 ± 0.2 s^-1^.mM^-1^) was similar to that of *P. ostreatus* VP1 (2.3 ± 0.2 s^-1^.mM^-1^) (see Table 1), although kinetic differences exist between the two enzymes. Likewise, when RB5 oxidation by the MnP6 variant was analyzed at lower pH, there was an increase of the catalytic efficiency; it was 14-fold higher at pH 2.5 than at the usual pH 3.5 (Table 2, bottom). In this case, the main improvement was related to the ninefold decrease of *K*_m_. More interestingly, when the kinetics at optimal pH are compared, the S168W variant of *C. subvermispora* MnP6 is over threefold more efficient than *P. ostreatus* VP1 in oxidizing RB5 (see Table 1), resulting in a lower *K*_m_ value (the two *k*_cat_ values are identical).

**Table 2:** **Kinetic constants for VA and RB5 oxidation by the S164W variant of** ***C. subvermispora*** **MnP6 at acidic pH values**

		pH 1.6	pH 2.0	pH 3.0
VA	*K*_*m*_ (μM)	650 ± 97	913 ± 52	2740 ± 560
	*k*_cat_ (s^-1^)	1.35 ± 0.06	1.08 ± 0.01	0.54 ± 0.04
	*k*_cat_/*K*_*m*_ (s^-1^ **.**mM^-1^)	2.08 ± 0.24	1.08 ± 0.06	0.197 ± 0
		**pH 2.5**	**pH 3.5**	
RB5	*K*_*m*_ (μM)	1.4 ± 0.2	12.6 ± 3	
	*k*_cat_ (s^-1^)	12.9 ± 0.7	7.8 ± 1.1	
	*k*_cat_/*K*_*m*_ (s^-1^ **.**mM^-1^)	9050 ± 930	619 ± 27	

Means and 95% confidence limits from triplicate measurements of substrate oxidation at 25°C in 100 mM BR buffer of different pH values.

### Lignin model dimer degradation and effect of pH

VA is the simple nonphenolic substrate used to estimate both LiP and VP activities in white-rot fungi. Therefore, VA oxidation by the S168W and S168W-environment variants already suggested the lignin-degrading ability of these enzymes. To confirm this, a nonphenolic β-*O*-4' lignin model dimer (4-*O*-methylsyringylglycerol-β-guaiacyl ether) was treated (at pH 3.0) with the S168W variant, which showed the highest efficiency and the most acidic pH optimum, oxidizing VA. Oxidative degradation of the model dimer occurred, showing that the variant was able to cleave the C_α_-C_β_ bond releasing 3,4,5-trimethoxybenzaldehyde.

The kinetic constants for the oxidative degradation of the dimer were estimated at pH 3.0 and pH 1.6 (Table 3) and compared with those obtained for *P. ostreatus* VP1 at pH 3.0 (this enzyme is quickly inactivated at more acidic pH values, as shown in Figure 1). As in the case of VA, oxidation significantly increased at more acidic pH, and the catalytic efficiency of the S168W variant oxidizing the dimer was sixfold higher at pH 1.6 than at pH 3.0. Although a small increase in turnover was observed when the reaction pH was lowered, the increased efficiency was mainly related to a lower *K*_m_ value (decreasing from 4.9 ± 1.3 μM to only 1.1 ± 0.2 μM). The latter *K*_m_ was 1.5-fold lower than that found for dimer oxidation by the *P. ostreatus* VP1 (Table 3) and, due to this difference, the VP-type engineered variant will be 1.5-fold more efficient at degrading lignins than natural VP, if the reaction is performed at the optimal pH of each enzyme.

**Table 3:** **Kinetic constants for degradation of a lignin model dimer by the S168W variant of** ***C. subvermispora*** **MnP6 (pH 1.6) and** ***P. ostreatus*** **VP1 (pH 3.0) (S168W oxidation at pH 3.0 is also shown)**

		S168W	S168W	VP1
		pH 1.6	pH 3.0	pH 3.0
Lignin dimer	*K*_*m*_ (μM)	1.1 ± 0.2	4.9 ± 1.3	1.7 ± 0.1
	*k*_cat_ (s^-1^)	0.47 ± 0.0	0.36 ± 0.1	0.49 ± 0.0
	*k*_cat_/*K*_*m*_ (s^-1^.mM^-1^)	0.42 ± 0.0	0.07 ± 0.0	0.28 ± 0.0

Means and 95% confidence limits from triplicate measurements of 4-*O*-methyl-syringylglycerol-β-guaiacyl ether degradation at 25°C in 100 mM BR buffer of pH 1.6 (only S168W) and pH 3.0.

## Discussion

### Catalytic promiscuity and acidic stability of lignin-degrading peroxidases

Among ligninolytic peroxidases produced by white-rot fungi, VP is arousing great interest because of its reaction promiscuity, combining the catalytic properties of the previously described LiP and MnP. Recently, Fernández-Fueyo *et al.* [17] demonstrated that *P. ostreatus* VP degrades a nonphenolic lignin model dimer and depolymerizes synthetic lignin as reported for *P. chrysosporium* LiP [7, 23], and suggested that VP in some Agaricales would play the same role as that of LiP in many Polyporales. Indeed, VPs can directly oxidize many other recalcitrant molecules, which LiP only oxidizes in the presence of VA [15, 24].

Due to the relatively low acidic stability of the above peroxidases, lignin depolymerization and other reactions are often carried out at less acidic pH conditions in which the enzymes do not present the maximal ligninolytic activity but are fully stable in time [17]. With the aim of avoiding such reduction in enzyme activity, in favor of maintaining the enzyme stability, we evaluated 14 white-rot fungal peroxidases with respect to acidic pH stability, and the so-called extralong and long MnPs [5] were found to be significantly more stable than the short MnPs and VPs. Among them, one extralong MnP (isoenzyme MnP6) from the *C. subvermispora* genome [25] maintained over 80% of the initial activity after 24-h incubation at pH 2.0, which was the highest acidic stability reported to date for a fungal member of the classical superfamily of non-animal peroxidases [17]. The presence of an extra disulfide bond and/or other interactions of the C-terminal tail in long and extralong peroxidases could contribute to this enhanced pH stability, since the acidic pH stability of *C. subvermispora* MnP6 was lost when the extra tail was removed (unpublished result).

### Engineering a VP-type peroxidase stable at highly acidic pH

Due to the stability characteristics described above, the *C. subvermispora* extralong MnP6 was used as a protein scaffold to engineer an acidic-pH stable VP-type peroxidase. Two previous studies described the introduction of the ability to oxidize VA in a MnP from *P. chrysosporium* [26] and in a generic peroxidase from *Coprinopsis cinerea* (synonym: *Coprinus cinereus*) [27]. Proceeding further in this direction, we aimed to engineer a peroxidase with wide (VP-type) substrate specificity on aromatics and dyes, and high acidic stability, two properties of interest for its use in enzymatic biocatalysis.

To expand the substrate specificity of the extralong MnP6 maintaining its stability, two variants were prepared after comparing its recently solved crystal structure (unpublished) with those of *P. ostreatus* VP1 [17] and *P. chrysosporium* LiP-H8 [28]. The S168W variant incorporated a putative catalytic tryptophan substituting the homologous Ser^168^, and maintained the original environment of this residue. In the S168W-environment variant, the environment of the new tryptophan was engineered to mimic the environment of the catalytic tryptophan in VPs [17] by introducing seven additional mutations, resulting, among other changes, in the substitution of three bulky phenylalanine residues located at the protein surface near the engineered Trp^168^.

The two variants incorporated to different extents the capabilities to oxidize both VA and RB5, which are characteristic of VPs [15]. When activities were estimated at the standard pH (3.0 to 3.5), the efficiency oxidizing VA was ≤10% of that found for *P. ostreatus* VP1, while the RB5 oxidation efficiency attained 55% of that estimated for VP1. This comparison also confirmed that LiP is more efficient at oxidizing VA, but has no activity on RB5. Interestingly, a comparison of the two variants revealed that the peroxidase with the VP-type environment of Trp^168^ has the highest efficiency oxidizing RB5, while the opposite happens for VA oxidation. On the other hand, the introduction of Trp^168^ did not significantly affect the kinetics for Mn^2+^ oxidation, and the extremely high efficiency of extralong MnPs in oxidizing the cation was largely maintained in the two VP-type variants (over 6,000 s^-1^.mM^-1^).

### Influence of the catalytic tryptophan environment

Residues surrounding the catalytic tryptophan would play a role modulating the substrate specificity and the efficiency of the reactions catalyzed, and could explain differences between LiP and VP reactions. LiP presents a more acidic tryptophan environment that would stabilize the VA cation radical [29], but could interfere with oxidation of anionic substrates, such as RB5. Ruiz-Dueñas *et al.* [30] demonstrated that the introduction of extra acidic residues in the tryptophan environment of VP from *P. eryngii*, emulating the LiP environment, results in the lack of direct RB5 oxidation, although a VA-mediated oxidation was possible, as found for LiP-H8.

The S168W variant presents a tryptophan environment inherited from the parent *C. subvermispora* MnP6 (Figure 3C) with only one exposed acidic residue (Glu^165^ equivalent to Glu^161^ and Glu^168^ in VP and LiP, respectively) and none of the basic residues present in the VP. The lack of additional acidic residues in the Trp^168^ surface environment could explain its low efficiency in oxidizing VA, and favor oxidation of RB5. The R257D mutation in the region of the catalytic Trp^164^ of VP from *P. eryngii* removes RB5 oxidation, whereas the R257A mutation does not affect this ability [30]. In agreement with these findings, the ability of the S168W variant to oxidize RB5 could be related to the absence of an acidic residue at this position, which in *C. subvermispora* MnP6 is occupied by Ser^272^. In the case of the S168W-environment variant, the RB5 catalytic constants were closer to those of VP (with an identical *K*_*m*_ of 5.4 μM). This could be due to the introduction of two basic residues at the Trp^168^ environment (Lys^268^ and Arg^272^), similar to those found in *P. ostreatus* VP1 (Lys^253^ and Arg^257^) (Figure 3A and C). Conversely, the presence of a more basic tryptophan environment in this variant would decrease the VA oxidation activity, which, as reported for LiP [29], can be improved by the presence of neighbor acidic residues stabilizing the aromatic cation radical. None of the MnP6 variants were able to oxidize DMP, suggesting that the engineered sites were unable to properly bind this phenolic substrate (opposite to nonphenolic VA). Finally, while a second set of kinetic constants could be determined for ABTS oxidation by VP1 (corresponding to a second low-efficiency oxidation site), this was not the case for the S168W variant, revealing that it oxidizes ABTS only at the catalytic tryptophan introduced by directed mutagenesis.

### Improved activity and enzymatic ligninolysis at acidic pH

It is well known that the oxidative activity of heme peroxidases increases at acidic pH. Gazarian *et al.* [31] described a tobacco peroxidase that oxidizes VA at pH 1.8, although it maintained the activity only for 15 minutes, and similar activities for shorter time periods have been reported for other plant peroxidases [32]. Some of these enzymes present a conserved tryptophan, but its eventual involvement in catalysis is yet to be demonstrated [33]. On the other hand, some palm peroxidases are extremely resistant enzymes [34] with acidic-pH stabilities comparable to those of the *C. subvermispora* extralong MnPs, but oxidation of high redox potential substrates (such as VA) has not been reported.

The two *C. subvermispora* MnP6 variants (S168W and S168W-environment) were highly stable at pH 2.0 (and showed only slightly lower residual activity at pH 1.6). Moreover, the optimal pH values for oxidation of VA (pH 1.6) and RB5 (pH 2.5) by the S168W variant (not by the S168W-environment variant) are lower than those found for *P. ostreatus* VP1 (pH 3.0), an interesting improvement considering that enzymatic ligninolysis is favored by acidic pH. Whereas the RB5 maximal activity of the S168W variant is at pH 2.5, suggesting that RB5 must be partially deprotonated for optimal binding, its activity on VA (a simple lignin model compound) progressively increases as the pH decreases. Since the two variants have similar acidic stabilities, their different pH optima must be due to the presence in one of them of a tryptophan environment mimicking that of the *P. ostreatus* VP, which resulted in optimal activities around pH 3, as found for VP. Moreover, the catalytic efficiency oxidizing VA and RB5 strongly increased (10- to 15-fold) when the kinetic constants were estimated at the optimal pH values. Under these conditions, the S168W variant is as efficient as the *P. ostreatus* VP1 at oxidizing VA and, interestingly, fourfold more efficient at oxidizing RB5.

The ability of LiP and VP to degrade lignins has been shown using nonphenolic lignin model dimers, in the absence of mediators [17, 23]. In contrast, MnP can only oxidize minor phenolic units in lignins, or phenolic lignin compounds. The introduction of a catalytic tryptophan in the extralong MnP from *C. subvermispora* confers the enzyme ability to oxidize a nonphenolic dimer, confirming the tryptophan functionality in enzymatic ligninolysis. Moreover, the potential of the S168W variant for lignin degradation is strongly enhanced by its acidic pH stability (50% residual activity at pH 1.6) and optimal activity (optimal VA oxidation at pH 1.6), enabling one to perform the reaction under more acidic conditions. In this way, a sixfold improvement in catalytic efficiency oxidizing the nonphenolic dimer was obtained when the reaction pH was lowered from pH 3.0 to pH 1.6, attaining efficiency values that were higher than those found for the *P. ostreatus* lignin-degrading VP1.

## Conclusions

A single mutation (S168W) added the ability to oxidize VA to an acidic-pH stable extralong MnP without significantly affecting its high Mn^2+^ peroxidase activity and only slightly lowering its remarkable acidic stability. Due to these new catalytic properties, together with its high intrinsic stability and the acidic optimal pH, the new variant behaves as a remarkably acidic-pH stable VP showing higher catalytic efficiency in oxidizing a non-phenolic lignin model dimer (VA and Mn^2+^) than the best native VP from the *P. ostreatus* genome. Moreover, the aromatic substrate promiscuity of this variant could be improved when the environment of the catalytic tryptophan was engineered by seven additional mutations resulting in a second variant, whose activity on RB5 (a recalcitrant molecule that cannot be oxidized by LiP) was several fold higher than that found for the best VP, when the reactions were performed at the optimal pH values. The results obtained demonstrate the possibility and interest of engineering ligninolytic peroxidases to obtain enzymes stable and active at extremely acidic conditions, resulting in enhanced oxidative power on the recalcitrant lignin polymers. For industrial utilization, these improved enzymes should be produced in heterologous expression hosts for their use as biocatalysts, or combined with other optimized hydrolytic (and oxidative) enzymes in a lignocellulose microorganism using a synthetic biology approach.

## Materials and methods

### Peroxidase coding sequences

The mature protein coding sequences of three VP (VP1 to VP3) and six short MnP (MnP1 to MnP6) genes from the *P. ostreatus* genomes (models 137757 from the monokaryon PC9 genome and 1113241, 156336, 1096331, 199510, 1089546, 1099081, 199511, and 1041740 from the monokaryon PC15 genome, respectively), five MnP (long MnP5, extralong MnP6, long MnP10, extralong MnP12, and short MnP13) genes from the *C. subvermispora* genome (models 49683, 50686, 117436, 157986, and 124076, respectively) and the LiP-H8 coding sequence from the *P. chrysosporium* genome (model 131707), together with the S168W-environment variant from *C. subvermispora* MnP6 (which contains the T162S, S168W, F258E, F262M, F268K, Q271N, S272R, and S275G mutations), were synthesized by ATG:biosynthetics (Merzhausen, Germany) after verifying that all the codons had previously been used for expressing other genes in the same *Escherichia coli* strain, and substituting them when required. The VP1 model (1089895) from the *P. ostreatus* PC15 monokaryon was discarded because of the presence of a premature termination codon, and it was therefore substituted by the PC9 allele (137757).

### Directed mutagenesis

The S168W variant was obtained by site-directed mutagenesis of the *C. subvermispora* MnP6 encoding gene by polymerase chain reaction (PCR) using the expression plasmid pET23a-50686 (see below) as a template, and the QuikChange™ kit from Stratagene. The 5'- G CCC CAG GAC AAT GTC ACA *TGG* ATC CTG GAG CGC- 3' direct primer (mutated codon in italics) and the reverse primer bearing the complementary sequences were synthesized. The PCR reaction (50-μl volume) was carried out in a Mastercycler proS thermal cycler (Eppendorf) using 20 ng of template DNA, 500 μM each dNTP, 125 ng direct and reverse primers, 2.5 units of *Pfu*Turbo polymerase (Stratagene), and the manufacturer buffer. Reaction conditions included i) a first cycle at 95°C (1 min), ii) 18 cycles at 95°C (50 s), 55°C (50 s), and 68°C (10 min), and iii) a final cycle at 68°C (10 min).

### Heterologous expression

The nine *P. ostreatus*, the five *C. subvermispora*, and the single *P. chrysosporium* peroxidase sequences, together with the two *C. subvermispora* MnP6 mutated sequences (S168W obtained by directed mutagenesis and S168W-environment obtained by DNA synthesis), were cloned into the expression vectors pFLAG1 (International Biotechnologies Inc.) or pET23a (+) (Novagen), and the resulting plasmids (pET23a-137757, pFLAG1-1113241, pET23a-156336, pFLAG1-1096331, pFLAG1-199510, pFLAG1-1089546, pET23a-1099081, pFLAG1-199511, pET23a-1041740, pET23a-49683, pET23a-50686, pET23a-50686/S168W, pET23a-50686/S168W-environment, pET23a-117436, pET23a-157986, pET23a-124076, and pFLAG1-131707) were used for expression.

The peroxidases were produced in *E. coli* BL21(DE3)pLysS (those cloned into pET23a) or *E. coli* W3110 (those cloned into pFLAG1). Cells were grown for 3 h in Terrific Broth, induced with 1 mM isopropyl-β-D-thiogalactopyranoside, and grown further for 4 h. The apoenzymes, which accumulated in inclusion bodies, as shown by SDS-PAGE, were solubilized with 8 M urea. Subsequent *in vitro* folding was performed using 0.16 M urea, 5 mM Ca^2+^, 20 μM hemin, 0.5 mM oxidized glutathione, 0.1 mM dithiothreitol, and 0.1 mg/ml protein, at pH 9.5 [17]. Refolding of *P. chrysosporium* LiP-H8 was performed as previously described [35]. Active enzymes were purified by Resource-Q chromatography using a 0 to 300 mM NaCl gradient (2 ml.min^-1^, 20 min) in 10 mM sodium tartrate, pH 5.5, containing 1 mM CaCl_2_.

### Kinetic constants

Steady-state kinetic constants for oxidation of representative substrates were estimated for the *E. coli*-produced native (wild-type) MnP6 from *C. subvermispora*, its S168W and S168W-environment variants, *P. chrysosporium* LiP-H8, and *P. ostreatus* VP1. Absorbance changes during substrate oxidation in 0.1 M tartrate (at various pH values) were recorded at 25°C in a Biomate 5 (Thermo Spectronic) spectrophotometer (using about 0.01 μM enzyme), with initiation by H_2_O_2_ (0.1 mM) addition. Oxidation of Mn^2+^ was followed at pH 5.0 by monitoring Mn^3+^.tartrate complex (ϵ_238_ 6.5 mM^-1^.cm^-1^) formation. VA oxidation and 4-*O*-methylsyringylglycerol-β-guaiacyl ether (nonphenolic lignin model dimer) oxidative cleavage were followed at pH 3.0 for veratraldehyde (ϵ_310_ 9.3 mM^-1^.cm^-1^) and 3,4,5-trimethoxybenzaldehyde (ϵ_310_ 6.3 mM^-1^.cm^-1^) formation, respectively. RB5, ABTS, and DMP oxidation were assayed at pH 3.5, and monitored for RB5 disappearance (ϵ_598_ 30 mM^-1^.cm^-1^) and formation of ABTS cation radical (ϵ_436_ 29.3 mM^-1^.cm^-1^) and dimeric coerulignone (ϵ_469_ 55 mM^-1^.cm^-1^), respectively. Means and standard errors for apparent affinity constant (*K*_m_) and enzyme turnover (*k*_cat_) values were obtained by nonlinear least-squares fitting to the Michaelis-Menten model. Fitting of these constants to the normalized equation *v* = (*k*_cat_/*K*_m_)[S]/(1 + [S]/*K*_m_) yielded the catalytic efficiency values (*k*_cat_/*K*_m_) with their corresponding standard errors.

### pH stability and optimal pH estimation

The pH stability was estimated by preincubating the purified enzymes (about 0.05 μM) in BR buffer at different pH values (pH 1.6 to 8.0). Residual activities were estimated by oxidation of saturating concentrations of ABTS (5 mM) or Mn^2+^ (1.5 mM) in 0.1 M tartrate (pH 3.5 or 5.0, respectively), under the standard conditions described above, immediately after mixing and after 4-h and 24-h incubation at 4°C. For each enzyme, the highest activity after mixing (at any pH) was taken as 100% activity, and the percentages of residual activity at the different times and pH conditions were calculated according to this maximal value.

The optimal pH values for substrate oxidation by the two *C. subvermispora* MnP6 variants and the native *P. ostreatus* VP1 were determined by measuring the oxidation of saturating concentrations of RB5 (15 μM) and VA (6 mM) in 0.1 M BR buffer of pH 1.6 to 5.0, as described above.

## Abbreviations

ABTS: 2,2'-azino-bis(3-ethylbenzothiazoline-6-sulfonate)
DMP: 2,6-dimethoxyphenol
LiP: lignin peroxidase
MnP: manganese peroxidase
RB5: Reactive Black 5
SDS-PAGE: sodium dodecyl sulfate-polyacrylamide gel electrophoresis
VA: veratryl alcohol
VP: versatile peroxidase.
